# Attitudes towards hearing difficulties, health-seeking behaviour, and hearing aid use among older adults in Thailand

**DOI:** 10.3389/fdgth.2023.1075502

**Published:** 2024-01-10

**Authors:** Panicha Pornprasit, Nattawan Utoomprurkporn, Poonsub Areekit, Pornnapat Manum, Sutatta Thanutammakul, Bhavisha Parmar, Joy Adamson, Doris Bamiou

**Affiliations:** ^1^ Faculty of Psychology, Chulalongkorn University, Bangkok, Thailand; ^2^ Faculty of Medicine, Chulalongkorn University, Bangkok, Thailand; ^3^ Sound Lab, Department of Clinical Neurosciences, University of Cambridge, Cambridge, United Kingdom; ^4^ Faculty of Brain Science, UCL Ear Institute, University College London, London, United Kingdom; ^5^ Department of Health Science, University of York, York, United Kingdom; ^6^ NIHR Biomedical Research Centre Hearing and Deafness, London, United Kingdom

**Keywords:** hearing aids, audiology, hearing loss, health seeking behaviour, stigma awareness, older adult health

## Abstract

**Introduction:**

This qualitative study explores older adults' attitudes towards hearing difficulties, health-seeking behaviour and hearing aid use in Samutprakarn, Thailand.

**Method:**

Three focus groups (*n*=25), with adults aged 60-88 years, were conducted within a residential complex for older adults. Qualitative data analysis, employing a phenomenological approach was completed.

**Results:**

Four themes are presented: (1) Perception of hearing difficulties, (2) Experience of receiving care, (3) Attitudes towards wearing hearing aids, and (4) Raising awareness of hearing difficulties in older adults. Findings indicate that Thai older adults may not be aware or accepting of their hearing difficulties, due to the stigma associated hearing loss, older age, and disability. The consequential impact of these unacknowledged issues manifest in communication challenges and the adoption of avoidance behaviours across diverse situational contexts.

**Conclusion:**

This study enhances our understanding of how Thai older adults perceive hearing problems and ear care. Findings underscore the potential pivotal role of familial and social networks in mitigating barriers associated with hearing difficulties among older adults in Thailand. These insights can raise awareness and inform effective management for older adults and their families. Additionally, the findings could guide healthcare providers, researchers, and policy makers, fostering improved care for this demographic.

## Introduction

The World Health Organization (WHO) estimates that 1 in 5 people experience disabling hearing loss. The prevalence increases to 2 in 3 people when evaluated in the populations aged over 60 years. Hearing difficulties affect 12.7% of adults aged 60–64 years of age, and this rises to 43.6% of those aged 80–84 years (1). The severity of hearing difficulties is associated with having a hearing handicap, with self-reported communication difficulties and social disengagement (2, 3). Recent meta-analysis showed that hearing loss is the highest modifiable risk factor for developing dementia (4). Hearing loss may also be used as an index for further cognitive decline (5).

Timely intervention of hearing loss may reduce dementia risk (6). Therefore, early hearing screening among the population can be beneficial and cost-effective (6). A recent WHO hearing report recommends hearing screening for people aged 50 and above (1). Previous research supported the idea that active screening is warranted since older adults usually wait for over 10 years after experiencing hearing difficulties before seeking medical help and receiving hearing aids (1). In addition, the long-term use of hearing aids was low, unless their hearing loss was more significant (6).

Establishing hearing screening programmes in the general population is challenging, due to factors involving poor awareness, access, high cost and the lack of associated intervention systems (7). Attitudes toward hearing difficulties and social stigma are also significant determinants preventing older adults from accessing hearing healthcare programmes (8). A systematic review showed that a self-perceived hearing problem was the most important factor influencing hearing-aid adoption (9). In Asian culture, many older adults and their families feel that hearing loss is a normal part of the ageing process, and interventions may not be considered as necessary (10). Some also oppose hearing intervention with hearing aids due to social stigma. A hearing aid is sometimes viewed as a burden and a sign of disability and ageing that might make others treat them differently (8).

Approximately 80% of people with hearing loss in developing countries do not receive hearing care (1). Thailand is one of few developing countries in Asia that offer hearing aids as part of the universal health care coverage. This means the patients do not need to pay for their hearing assessment and evaluation including intervention from doctors and healthcare professionals. Even though older adults are eligible to reimburse hearing aids without any additional cost, many are still not receiving hearing screening and intervention (8). Previous qualitative studies have investigated hearing healthcare service users' perspectives of audiology care to identify factors that could improve services and hearing aid use. However, this data has not captured the perspectives of older adults from low-resource contexts (11). A study in Malawi found that hearing aid uptake was low, even though the device was offered free of charge (12). It is therefore particularly important to explore the attitudes and experiences of people in the wider population as this may uncover specific unique cultural/belief systems or societal barriers that could impact hearing aid adoption.

This study aims to explore older adults’ attitudes toward hearing difficulties, health-seeking behaviour and hearing aids use in Thailand. Understanding their attitudes and behaviour could help the government, public health authorities, researchers and collaborators work together to create better policies to support their acceptance of hearing screening and intervention. Moreover, gathering this information could shape a better dialogue to promote and raise awareness about the importance of hearing screening and intervention among older adults, their families, and care givers.

## Material and method

### Ethical approval

All materials and study procedures were approved by the institutional review board at Chulalongkorn University under the Faculty of Psychology section. All participants gave written consent to take part in the study. No participant-identifiable data was recorded or presented.

### Grant

Ratchadapiseksompotch Fund, Faculty of Medicine, Chulalongkorn University, grant number RA-MF-14/66, RA-MF-02/66 and GA-66/15.

### Study design

This study used a descriptive and explorative, qualitative design. The recorded focus group exchanges were transcribed verbatim for the analysis.

### Participants

The participants were residents at Sawangkanives, an older adult apartment complex in Samutprakarn, Thailand under the care of the Thai Red Cross organization. The advertisement posters about the activity were distributed in the community about 1 week before the interview. Twenty-six participants (4 male, 22 female) aged from 60 to 88 years (mean age = 77.73 years, S.D. = 8.11), took part in the focus groups. Participants were included in the present study if they were aged 60 or older and able to communicate verbally.

### Focus group protocol

The participants were divided into three groups (group 1: *n* = 7, group 2: *n* = 9, and group 3: *n* = 10) and the research activity was conducted on July 22, 2022, at the activity rooms of Sawangkanives, by the members of the local research team. Since this study includes a range of participants who belong to a similar age group and live in the same community, a focus group methodology was adopted to encourage interactions, discussions, and sharing experiences among older adults (13). A topic guide of semi-structured questions was developed specifically for this study to capture the experience of hearing difficulties, health-seeking behaviour, and hearing aid use in our focus groups (Table 1). The questions were developed collaboratively within our research team consisting of psychologists with experience working with older adults, audiologists, ear nose throat doctors and staff of the apartment complex.

**Table 1:** Focus group semi-structured interview schedule.

Hearing difficulties
• Do you have hearing difficulties? If yes, how do you experience hearing difficulties? • How do hearing difficulties affect your living?
Receiving care
• When you realize you have hearing difficulties, what did you do about it? • Why did you decide to get treatment? • What do you think about the treatment of hearing difficulties?
Hearing aids
• Do you use hearing aids in daily life? • What is the effect of using hearing aids? • How do you think about hearing aids?
Hearing difficulties knowledge
• How do you get information about hearing difficulties treatment? • Which kind of publicity about hearing problems will reach you the most and how?

The focus group discussions centred on eliciting participants' attitudes toward hearing difficulties and their lived experiences of hearing loss. Participants were prompted to share narratives detailing their personal encounters with hearing challenges and to articulate their experiences of incorporating hearing aids into their daily routines. Each focus group discussion spanned a duration of one hour. To capture the discussions comprehensively, voice recorders were employed, and subsequent verbatim transcriptions were recorded.

### Data analysis

Qualitative analysis with a phenomenological approach was used to focus on participants' lived experiences of hearing difficulties and hearing aid use. This method was chosen since it identifies and characterizes the meaning and understanding of a particular phenomenon with depth (14, 15).

Once the transcripts were read thoroughly, initial codes were generated. Codes were then grouped into themes and consensus was achieved between researchers to ensure consistency in interpretation. The researchers reflected on the themes to develop a structural description of the underlying meaning of an experience for a particular group of people.

## Results

As a result of data analysis, four main themes were identified: (1) Perception of hearing difficulties, (2) Experience of receiving care, (3) Attitudes towards wearing hearing aids, and (4) Raising awareness of hearing difficulties in older adults.

### Perception of hearing difficulties

Participants described their specific experiences of hearing difficulties and how hearing loss impacted their communication, interactions, and speech perception. Some discussed whether their previous ear trauma, recurrent otitis media, viral infection, or talking on the phone (while it was charging), was associated with hearing loss. The majority noticed changes to their hearing perception, as speech became less clear, resulting in conversation breakdown.

*I realize when others speak, I have to ask “what, what, what?”, approach them, and tell them to repeat in a louder voice. At the normal sound level, I can hear the sound, but I cannot understand it. (Female, 87 years, group 1)*

*Many years ago. As I said, why do I hear the sound, but I can't hear them as words. This senior said that (the sound in her ear) was music but mine was some sort of sound I could not describe. But as I said, that's it. That's the way it is. I don't know for how many years, but it's been a long time. (Female, 72 years, group 3)*

*I knew when I couldn't catch the words, I could only hear sounds. Sometimes I could not catch the words. I didn't know what was being said. (Female, 84 years, group 3)*

There was a range of hearing losses and device use within the cohort. Some participants wore hearing aids, some had hearing loss diagnosed but did not wear hearing aids, and others did not have a diagnosed hearing loss. A few participants mentioned that their friends, family and health professionals had first made them aware of their hearing problems.

*It is because my wife complained that I had hearing loss and couldn't hear what she was saying. (Male, 81 years, group 1)*

*I don't know anything. I went to another doctor. The doctor there told me that I will be sent to this (hearing) department. And so, I went where the doctor sent me. (Female, 88 years, group 3)*

Hearing problems affected daily life in a variety of ways. Most of the reported difficulties impacted social communication and interpersonal relationships, and this sometimes led to avoidance behaviours.

*Bad ears can't get along with anyone. I became a loud talker. Though friends would tell me that it's a small matter, just talk in a softer volume. (Female, 65 years, group 2)*

*I would avoid the conversation, but the thing is: I want people who talk softly to me to speak louder. One way that could make them speak louder is to tell them directly to speak louder. I tried, and they didn't. So, I stopped (using this method). So, I became a loud talker. Nowadays I speak loudly. In the past, when I was young, I used to speak in a soft voice. Now, whatever group I have conversations with, I speak loudly. (Female, 87 years, group 2)*

*It's because of this: others speak, and I can't hear. So, I quit. Sometimes I quit calmly. For instance, if it's the TV, I just turn it off. And then I listen to what I want, turn on stuff, and live my life. But I still want to get better. I want it to disappear. (Female, 80 years, group 2)*

### Experience of receiving care

Here, participants described how and when they decided to seek help with their hearing. They reported how hearing difficulties in specific day-to-day activities prompted them to visit healthcare providers for support.

*I work. If somebody said something, I would not be able to catch what they were saying. Similar problem, I can hear but I cannot make out the words, so I wear (hearing) aids. (Female, 79 years, group 3)*

*I was sitting in the backseat of a car with my grandchild. I could hear the people in the front talking but I couldn't catch what they were talking about. I told my grandchild, so he took me to the doctor. (Female, 83 years, group 3)*

Others were prompted to seek further medical advice by their friends or family members, or they were taken to the doctor by their family members (including their wife, children, or grandchildren).

*My wife complained that I had hearing loss and couldn't hear what she was saying, so I must go. (Male, 81 years, group 3)*

*My ears were stuffy, and I had vertigo. People said I had an ear problem, so I went to get an ear and hearing checkup. (Female, 71 years, group 3)*

*When I was 40 years old, I felt like I started to have hearing loss, so I consulted my friend. My friend told me to use hearing aids, so I got them reimbursed. (Female, 66 years, group 1)*

### Attitudes towards wearing hearing aids

Participants described a mixture of positive and negative feelings and experiences about wearing hearing devices. Factors influencing their opinions included practical usage of hearing aid devices, preconceived opinions of their appearance and how the wider societal perceptions.

*“My high school friend wore hearing aids and I noticed that he was burdened by turning it on and off, changing batteries, visiting the doctor, electricity, and the price.” (Female, years, group 1)*

*One, it doesn't work. Second, maybe it's annoying, I'm not sure. Third, if I don't need it, then I don't want to wear it. (Female, 83 years, group 1)*

The initial fitting of the hearing devices seemed to produce overwhelming sensations including dizziness and unnatural sounds which were difficult to acclimatize to.

*When I wore them, I felt dizzy and wobbly, so I didn't wear them. (Female, 85 years, group 3)*

*At first, I wasn't used to it. Because it's as if there's a microphone in the ear, I couldn't adapt to it. (Female, 79 years, group 3)*

*‥what I don't like is that it doesn't feel natural when I wear it. I want it to be like normal. (Female, 83 years, group 3)*

Some participants reported dissatisfaction with the aesthetics of hearing aids and its negative association with the ageing process and disability.

*In the past, hearing aids were big and ugly. It's embarrassing. (Female, years, group 1)*

*For me, I don't like wearing it because it makes me look like a disabled or an old person. Sometimes I don't understand what is said but I have to endure it. The exception is when I attend meetings. Normally I don't wear it. I don't like people viewing me as disabled or old. (Male, 76 years, group 3)*

Positive perspectives of hearing aid use were also reported. Some felt hearing loss was a natural part of ageing and therefore hearing aid use would be inevitable. They wanted people with hearing loss to wear hearing aids so that they would know how to adapt their communication accordingly. Their perception of presbycusis is a natural degeneration by age and wearing hearing aids is comparable to wearing glasses.

*I don't think I have to be embarrassed. Rather, I want to announce to people that I am wearing hearing aids. (Female, 88 years, group 3)*

*If I can't hear, then I will wear (hearing aids) because I want to be able to hear like normal people. People speak well but too soft. I feel pity. (Female, 78 years, group 2)*

*It's like wearing eyeglasses. It's the same. It's normal because we cannot hear clearly. (Female, 80 years, group 2)*

*I wear it and it doesn't matter whether I am viewed as old or young, because I am old.. (Female, 83 years, group 3)*

*I'd say this is normal. Because the human body, to put it simply, comparing it to, for example, a car that has been used for 10 years is already considered bad and needs replacement. Then what about our body, right? It is something fragile, how can it still survive? That's all. I thought about this, and I felt sympathetic. I am just worried whether they will be able to hear. With our body, I believe the elderly would understand that one day we might meet this end. (Female, 80 years, group 2)*

### Raising awareness of hearing difficulties in older adults

There was a lack of accurate knowledge and awareness about hearing loss and ear care within the group of Thai older adults. Participants recommended ways to improve this by involving family members.

*Like talking with children, it depends on their background and their education level. If they lack knowledge, then you have to give them a simple explanation from the basics. Make them understand the importance and the consequences of using and not using (hearing aids). Befriend and familiarize yourself with them. (Female, 79 years, group 2)*

*Be friendly and kind. Show them and they will slowly trust you (and your advice) (Female, 80 years, group 2)*

Besides communication with family members, educating the wider society can help increase awareness of hearing problems. Some participants suggested how social media and community services could be used to inform older adults of important health information.

*Approach via social media…, and they will share it on and on. (Female, 60 years, group 3)*

*Volunteers in community service might be able to help because the elderly may not have opportunities to visit the hospital. (Female, 80 years, group 3)*

## Discussion

Negative perceptions and attitudes towards hearing difficulties, health-seeking behaviour, and hearing aid adoption in Thai older adults can affect their access to hearing healthcare. Four main themes were presented in the present study: (1) Perception of hearing difficulties, (2) Experience of receiving care, (3) Attitude towards wearing hearing aids, and (4) Raising awareness of hearing difficulties in older adults. From the presented themes, we have created three takeaway messages as discussed below.

### Associating hearing loss with older age

Risk factors of presbycusis include genetic predisposition, hormones, exposure to loud noise or ototoxic agents, history of ear infection, and systemic diseases (16). In this study, we were not aware of the participants' baseline hearing before the onset of presbycusis, but it is possible that ear diseases could have existed in their earlier years. Despite noticing hearing difficulties in later life, participants tended to report different causes of hearing loss, rather than labelling it as age related alone. This may suggest a lack of acceptance of potential hearing changes associated with ageing. Often, the people around them (e.g., children and grandchildren) made them aware of their hearing difficulties. This is consistent with previous research which showed people close to the patient notice the hearing loss before the patient does (1).

Unaddressed hearing loss has a significant impact on the participants' lives. The typical presentation of presbycusis is difficulty discriminating speech, especially in situations with background noise. This is partly due to the impaired ability to understand high-frequency components of speech. Some patients also experience tinnitus, which is not specific to the disease (16). These symptoms negatively impacted daily activities, specifically understanding conversations with family and friends.

One of the barriers to the adoption of hearing aids in some individuals was the fear of becoming dependent on devices, and some participants did not want to have to wear hearing aids all the time. The desire for independence may be related to the construction of the self-concept of older adults. Literature found that aging (17) and disability (18) are associated with lower levels of self-concept. Presbycusis is associated with these two stigmas (8). Perceiving themselves as younger enhances self-esteem and mental health (17). Most participants in the present study expressed that they would wear a hearing aid if they have hearing loss. However, they also denied having a level of hearing loss that would require hearing aids. This study found the reasons behind the refusal to adopt hearing aids includes stigma towards hearing loss, physical appearance of hearing aids, care and maintenance, fitting problems, and technological limitations. Overall, the stigma towards hearing aids due to the physical appearance of hearing aids has decreased with its size over the years (19). However, there may be specific contextual factors that exacerbate stigma for the population included in this study.

In Thailand, the Universal Health Coverage Scheme requires an additional legal disability registration (20), which could enhance the stigma of being labelled as disabled. The denial of hearing problems and rejection to adopt hearing aids can be a defense mechanism (21). The visibility of a disability may have further effects on self-concept. A study found that individuals with invisible psychiatric disabilities had lower levels of self-concept and body image compared to individuals with visible physical disabilities (18).

### Hearing aid adoption: The need for family support

Consistent with previous research, participants in this study tended to wait years (range: 3 years to 10+ years) before seeking medical advice for their hearing loss (19). The decision to see the doctor was either due to realising their self-perceived hearing loss or following the advice of their significant others. Most participants decided to seek treatment because they experienced a higher degree of self-reported hearing loss and longer duration of hearing problems. This aligns with previous literature demonstrating that self-perceived hearing problems were the most important determinant of hearing aid adoption (9).

In Thailand, older adults are respected and cared for by their families (22). Filial piety, the obligation to assist, honour, and sacrifice for parents, is a common custom across many Asian cultures (23). Minimization of hearing problems is made worse by significant others adjusting to compensate for the hearing loss (21). As part of filial responsibility, which is common in low-middle income countries, elders live with younger family members who are obliged to speak more loudly or turn the TV volume up to avoid communication breakdowns. Since it is a typical occurrence that people close to patients are the first to notice hearing difficulties, raising awareness of hearing healthcare in families could improve health-seeking behaviour.

### Role of healthcare systems in enhancing hearing care

Healthcare providers play a significant role in the compliance to hearing aid usage. This may be due to the nature of paternalistic doctor-patient relationship in Thailand (24). The physician is authoritative and is viewed as the one with knowledge. A study on Thai diabetic patients and their adherence to medication suggests that patients with good compliance perceived more empathy (25). On the other hand, this power dynamic could be a barrier to patients with poor adherence disclosing failure to comply with the doctor's recommendations (25). Thus, effective communication with the patient may encourage hearing aid adoption.

Problems related to the initial fitting of hearing aids, including ear discomfort and dizziness, have discouraged hearing aid users from continuing to wear the device. Many of these could be resolved by accessing audiological counselling, care and rehabilitation (26). First time visits should also consist of training for practical usage, for instance insertion, removal, functions, and maintenance of hearing aids. Follow-up appointments can be scheduled every one year (19).

A long-term issue is the burden of hearing device care and maintenance. Although hearing services are included in universal health coverage, hearing devices require maintenance. Changing batteries could impose both mechanical and financial challenges (27). Previous studies also highlighted handling problems and volume control (19). Visits for hearing aid adjustments can be seen as a burden. Patients are faced with the technological limitations of hearing aids which provide poor benefit and unnatural sound or difficulty hearing speech in noisy environments. Hearing aids are able to amplify and compress sounds but fail to compensate for distortions in neural activity (28). Machine learning may have the potential to improve hearing aids performance (29).

Previous studies found financial reasons to be a prominent barrier to hearing aid use (8). Participants in this study did not report the cost of hearing care services to be a limiting factor, possibly because in Thailand, hearing aids services are included in all health benefit scheme. The largest health insurance scheme, Universal Health Coverage, was responsible for providing hearing aids for 16% of those with hearing loss (30). The proposed idea that public health insurance of each country could improve hearing aid adoption may not be the case. Similarly, the National Health Service in the UK enables access to hearing aids services, from batteries to repairs. In 2019, 9% of adults with hearing loss (aged 40 to 69 years old) adopted hearing aids (31). On the contrary, hearing aids are not included in the US Medicare program. The 2019 prevalence of hearing aid use in US adults older than 65 was 14.4% (32). The difference in age groups in these studies makes comparison between the prevalence of hearing aid adoption difficult. Nevertheless, these countries all have a common ground: the low uptake of hearing aids. Affordability may increase adoption (8), but it is not the only factor impacting hearing aid adoption and long-term usage.

Providing accurate knowledge and information to the public can address both stigmas of hearing loss associated with older age and with disability, which are a concern for older adults. Participants in this study proposed two methods in raising awareness of hearing difficulties in older adults: (1) direct communication with patients and family members and (2) sharing information using social media. Participants stressed that the way knowledge is relayed should be appropriate to the patient's educational/cultural/language background. Since they recognize that information should be provided to patients, caregivers, and family members, it is important to recognize that these groups may have different levels of knowledge and experience.

Participants also suggested that physicians should help patients to engage in conversations about hearing loss by being friendly, empathetic, and sincere. This reflects the audience-centered principle in public health communication (33). The WHO World Report on Hearing introduced two measures: education and hearing screening programs for different risk groups (1).

Our participants also proposed using social media for sharing information on healthcare. Previous studies report that Thai seniors actively use social media on mobile phones on a daily basis (34, 35). Line and Facebook were the most frequently used platforms. Although it is often used for entertainment, the Thai older adult community also use the internet to search for health-related information. Interest in online health information was influenced by the individual's beliefs and illness, which made up the “self” in Bandura's Social Cognitive Theory (34). Seniors were interested in the treatment of a certain disease only if they had that disease. After being exposed to online health media, many follow given recommendations, discuss the information with family and friends, search for more information, and share the information. Additionally, most older adults could discern between advertisements, fake news, and trustworthy health information. Referencing the name of the physician in the media was an important factor in increasing credibility (34). Previous study suggests that cognitive decline may not be a barrier to social media use in Thai older adults (35). Thus, social media could be a potential platform in providing accurate knowledge and raising awareness among older adults as long as the appropriate monitoring and evaluation is incorporated.

## Strengths, limitations and future research

This is one of the few qualitative studies exploring the experiences of people accessing hearing healthcare in developing countries. Our study was conducted in a setting where the cost of hearing care was not a barrier to service use, showing that affordability is not the only factor impeding hearing healthcare access.

There are some limitations within the current study. We did not collect data on self-reported hearing loss or hearing levels in the participant group, and therefore we were unable to explore whether experiences differed based on severity of hearing loss. Also, participants resided within a single older adult residential hall, and therefore may not have had wide-ranging experiences of healthcare access. Future research should focus on exploring how health-seeking behaviours of older adults in this population can be improved. This study suggests investigating methods of communication which could raise awareness of hearing health, such as distribution of accurate information through social media and family/community groups. Future studies could also explore if experiences of seeking and accessing hearing healthcare have changed over time.

## Conclusion

This study enhances our understanding of how Thai older adults perceive hearing problems and ear care. Findings underscore the role of familial and social networks in mitigating barriers associated with hearing difficulties. There is a need to raise awareness in the Thai older adult population, and within families and communities to improve access to hearing healthcare. These insights help guide healthcare providers, researchers, and policy makers, fostering improved care for this demographic.

## Data Availability

The raw data supporting the conclusions of this article will be made available by the authors, without undue reservation.
